# Brain white matter structure and information processing speed in healthy older age

**DOI:** 10.1007/s00429-015-1097-5

**Published:** 2015-08-09

**Authors:** Ksenia A. Kuznetsova, Susana Muñoz Maniega, Stuart J. Ritchie, Simon R. Cox, Amos J. Storkey, John M. Starr, Joanna M. Wardlaw, Ian J. Deary, Mark E. Bastin

**Affiliations:** Doctoral Training Centre in Neuroinformatics and Computational Neuroscience, School of Informatics, University of Edinburgh, Edinburgh, UK; Institute for Adaptive and Neural Computation, School of Informatics, University of Edinburgh, Edinburgh, UK; Brain Research Imaging Centre, Neuroimaging Sciences, University of Edinburgh, Edinburgh, UK; Scottish Imaging Network, A Platform for Scientific Excellence (SINAPSE) Collaboration, Edinburgh, UK; Centre for Cognitive Ageing and Cognitive Epidemiology (CCACE), University of Edinburgh, 7 George Square, Edinburgh, EH8 9JZ UK; Department of Psychology, University of Edinburgh, 7 George Square, Edinburgh, EH8 9JZ UK; Alzheimer Scotland Dementia Research Centre, University of Edinburgh, Edinburgh, UK

**Keywords:** Ageing, White matter structure, Information processing speed, Diffusion MRI

## Abstract

Cognitive decline, especially the slowing of information processing speed, is associated with normal ageing. This decline may be due to brain cortico-cortical disconnection caused by age-related white matter deterioration. We present results from a large, narrow age range cohort of generally healthy, community-dwelling subjects in their seventies who also had their cognitive ability tested in youth (age 11 years). We investigate associations between older age brain white matter structure, several measures of information processing speed and childhood cognitive ability in 581 subjects. Analysis of diffusion tensor MRI data using Tract-based Spatial Statistics (TBSS) showed that all measures of information processing speed, as well as a general speed factor composed from these tests (*g*_speed_), were significantly associated with fractional anisotropy (FA) across the white matter skeleton rather than in specific tracts. Cognitive ability measured at age 11 years was not associated with older age white matter FA, except for the *g*_speed_-independent components of several individual processing speed tests. These results indicate that quicker and more efficient information processing requires global connectivity in older age, and that associations between white matter FA and information processing speed (both individual test scores and *g*_speed_), unlike some other aspects of later life brain structure, are generally not accounted for by cognitive ability measured in youth.

## Introduction

Cognitive ageing is associated with decline in average levels of some cognitive capabilities, such as information processing speed, abstract reasoning, executive function and aspects of memory, even in the absence of overt pathology (Salthouse 2010). Understanding the biological bases of cognitive ageing is therefore an increasingly important area of research for developed societies with ageing populations (Deary et al. 2007, 2009). Brain structural changes are also commonly observed in normal ageing and include atrophy of both grey and white matter regions (Bartzokis et al. 2001; Raz et al. 2005), ventricular enlargement, white matter hyperintensities (WMH) in deep and periventricular regions (Fazekas et al. 1987), enlarged perivascular spaces (Doubal et al. 2010) and mineral deposition (Bartzokis et al. 2007). The present study focuses on cerebral white matter, and hence the brain’s ability to pass information efficiently between different cortical regions, an important factor in terms of understanding cognitive functioning in older age (Gunning-Dixon et al. 2009; Malloy et al. 2007; Penke et al. 2012).

It has been argued that information processing speed is a fundamental characteristic of the brain’s cognitive efficiency. This is because it is relatively elementary; the tests which assess it require only simple and repetitive responses to questions which would not attract errors if given sufficient time. Performance on simple tests of processing speed is associated with scores on more complex cognitive tests, such as reasoning (Jensen 2006). Processing speed might therefore mediate how age-related brain changes influence the efficiency of ‘higher’ cognitive functions (Deary 2000; Salthouse 1996; Verhaeghen 2014; Penke et al. 2012).

A number of cognitive tests with different stimulus–response arrangements can provide measures of processing speed. These include paper-and-pencil psychometric tests, such as Digit-Symbol Substitution (Wechsler 1998), as well as psychophysical measurements, such as Choice Reaction Time (Deary et al. 2001; Jensen 1987) and Inspection Time (Deary 2000; Deary et al. 2010). Performance on processing speed tests is correlated; subjects who are more efficient on one speed test tend to be more efficient on the others. Therefore, data reduction techniques, which have the advantage of removing error and task-specific variance associated with each individual test, can be used to combine an individual’s performance on several tests into a general index of information processing speed (which we name ‘*g*_speed_’). This is typically the first unrotated component (sometimes called a ‘factor’) from a principal component analysis (PCA) of scores from the individual tests which captures the largest proportion of the covariance between them (Luciano et al. 2009). Information processing speed, and hence *g*_speed_, decreases with age and may therefore provide a useful indicator of cognitive decline in later life (Deary et al. 2010; Ritchie et al. 2014; Salthouse 1996; Verhaeghen 2014).

Brain white matter structure in older age can be investigated using imaging biomarkers derived from diffusion tensor MRI (DT-MRI), such as fractional anisotropy (FA) and mean diffusivity (MD) (Salat et al. 2005; Sullivan and Pfefferbaum 2006). Although ascribing these biomarkers directly to white matter ‘integrity’ is problematic (Jones et al. 2013), a number of authors have used them to investigate links between brain structure and processing speed in older age. For example, Kennedy and Raz (2009) examined relationships between FA measured in several white matter regions and cognitive tests, including processing speed, in a sample of 52 subjects aged between 19 and 81 years. After correcting for the effects of age, only working memory and processing speed remained significantly associated with FA in anterior brain regions. Haász et al. (2013) investigated links between general fluid intelligence (*g*_f_) and white matter water diffusion parameters in a cohort of 100 healthy participants aged between 49 and 80 years. Using two measures of processing speed, a colour-word interference test and a visuo-spatial attention task, as well as several other tests to compute *g*_f_, they found associations between *g*_f_ and MD, FA and radial diffusivity across the white matter skeleton with Tract-based Spatial Statistics (TBSS; Smith et al. 2006). However, of all *g*_f_ tests, processing speed scores contributed most towards associations with white matter structure.

Vernooij et al. (2009) investigated relationships between brain white matter water diffusion parameters and cognitive ability in 860 people aged 60 and older. They found that elevated mean, axial and radial diffusivities and decreased FA were associated with worse performance on processing speed and general cognitive ability tasks for both normal-appearing white matter and that located within WMH. In their study of 554 adults aged 70–82 years, van den Heuvel et al. (2006) found that periventricular WMH were significantly associated with decreased processing speed performance as measured by the Letter-Digit Coding test.

Although these studies provide valuable insights into links between brain white matter structure and cognition in older age, they tend to include participants with a wide range of ages. This is problematic since chronological age is a major confounder of cognition and white matter structure, and age-heterogeneous cohorts may mask its true effect (Hofer and Sliwinski 2001); for example, white matter FA and MD are known to decrease and increase, respectively, with advancing age requiring mediation analyses to account for such effects in brain-cognition studies of older adults (Madden et al. 2012). In the present study, we investigate relationships between white matter structure and information processing speed using voxel-based methods in the Lothian Birth Cohort 1936 (LBC1936), a large group of generally healthy subjects in their seventies with a narrow age range. These attributes, coupled with the availability of a validated measure of cognitive ability from childhood and a diverse range of processing speed tests performed in older age, make this cohort valuable for studying associations between brain structure and cognition in older age.

Previous studies conducted in the LBC1936 have assessed associations between general factors of information processing speed (*g*_speed_), general intelligence (*g*) and brain white matter DT-MRI biomarkers measured in tracts segmented using quantitative tractography. Penke et al. (2010) reported associations between tract-average white matter FA, MD, axial and radial diffusivity, and *g*_speed_ in a pilot sample of the LBC1936 (*n* = 132). They found that *g*_speed_ had a significant association with tract-average FA and MD in all eight major tracts investigated (genu and splenium of corpus callosum, and bilateral cingulum cingulate gyri, uncinate and arcuate fasciculi). This suggests that *g*_speed_ is likely to be a property related to global brain connectivity rather than to specific tracts. Penke et al. (2010) also demonstrated that, like *g*, a general brain white matter FA factor (explaining 40 % of the individual differences across all eight tracts) could be extracted from the tractography data and this factor was associated with *g*_speed_ (*r* = −0.24, *p* = 0.007). In a further analysis conducted when all LBC1936 participants’ brain imaging data were available, Penke et al. (2012) studied the relationships between tract-average FA, longitudinal relaxation time and magnetisation transfer ratio with *g* and *g*_speed_ in 12 major white matter tracts in 420 subjects (the eight tracts measured in Penke et al. (2010) plus bilateral inferior longitudinal fasciculi and anterior thalamic radiations). General factors of all three tract-averaged biomarkers (explaining 40–70 % of the individual differences across all 12 tracts) were independently associated with *g* (together explaining 10 % of the variance) and, importantly, this effect was completely mediated by *g*_speed_. This is amongst the strongest evidence to date that processing speed is a mediator between brain white matter structure and general cognitive functioning. However, these previous studies have measured imaging biomarkers averaged across specific tracts, and have not investigated whether, and where, individual tests might be correlated with white matter structure using methods that allow greater spatial localisation of effects.

The present paper reports new analyses on the LBC1936, testing the extent to which white matter structure is related to differences in processing speed measures. For these analyses, we used TBSS rather than quantitative tractography methods. TBSS allows analysis of DT-MRI data to be conducted on a voxel-wise basis across the brain, therefore revealing more detail on the spatial distribution of associations between white matter structure and cognitive ability than is possible with quantitative tractography. We first tested white matter FA associations with both *g*_speed_ and the individual speed tests contributing to it. We also assessed whether specific aspects of the individual processing speed tests were associated with white matter structure beyond that accounted for by *g*_speed_ (Booth et al. 2013). Finally, since our participants had measures of their childhood intelligence available, we tested whether later life associations between white matter FA and processing speed were still present after the speed tests had been adjusted for earlier cognitive ability.

## Methods

### Participants

The LBC1936 comprises 1091 community-dwelling individuals in their seventies who agreed to participate in a longitudinal study of cognitive ageing (Deary et al. 2007). At age 11 years, almost all of them took part in the Scottish Mental Survey of 1947, which employed the Moray House Test No. 12, a test of general cognitive ability. This test, which takes 45 min to complete, is mainly one of verbal reasoning, but includes a variety of other items on areas such as spatial abilities, arithmetic and cypher decoding (Scottish Council for Research in Education 1949). At recruitment in older age, subjects agreed to further cognitive testing and other medical, physical and psychosocial assessments at approximately 70 years of age (Deary et al. 2007). During the second wave of this study, and in addition to follow-up cognitive and health testing, participants also underwent a comprehensive MRI examination, including DT-MRI, to assess brain white matter structure at approximately 73 years of age (Wardlaw et al. 2011; Deary et al. 2012). Study inclusion criteria for the present report were that participants should have complete DT-MRI, childhood intelligence and age 73 processing speed data. Participants were also asked a series of Yes/No questions on their medical history by an interviewer. The questions determined whether participants had a history of cardiovascular disease or high cholesterol, and were being treated for hypertension, diabetes or blood circulation problems. Written informed consent was obtained from all subjects under protocols approved by the UK National Health Service Ethics Committees.

### Tests of information processing speed

In the second wave of the study, and approximately contemporaneous with their brain MRI scan, subjects undertook a battery of cognitive tests designed to characterise a wide range of cognitive domains (Deary et al. 2007; Tucker-Drob et al. 2014). Processing speed was a particular focus of the study. Subjects undertook five processing speed tests, namely Digit-Symbol Substitution and Symbol Search from the Wechsler Adult Intelligence Scale, 3rd Edition (Wechsler 1998), Simple Reaction Time, Four-Choice Reaction Time and Inspection Time; these tests are described in Deary et al. (2007).

For Digit-Symbol Substitution, participants were given a set of digit-symbol transformations as a ‘dictionary’ and asked to decode as many symbols as they could in two minutes. In Symbol Search, participants indicated whether or not one of a pair of symbols occurred in a row of symbols. The number of correct answers within the allotted time (two minutes) was recorded and used for subsequent analysis. Both tests were administered according to the standard instructions in the Wechsler manual.

Mean and SD (intra-individual variability) values for Simple and Four-Choice Reaction Time were measured using a portable reaction time device designed for the UK Health and Lifestyle Survey (Cox, Huppert and Whichelow 1993; Deary et al. 2001). The box includes a high-contrast LCD display and five response keys labelled 1, 2, 0, 3, 4 arranged in a shallow arc. For Simple Reaction Time, participants were asked to rest a finger on the 0 button; once a 0 appeared on the screen, the participants were told to press the button as rapidly as they could. The test consisted of 8 practice and 20 test trials. In the Four-Choice Reaction Time task, participants were told to place the second and middle fingers of each hand on the buttons labelled 1, 2, 3 and 4. Participants were then presented with a number (1, 2, 3 or 4) on the LCD screen, and were told to press the corresponding button as quickly as possible. The test consisted of a total of 48 trials: 8 practice and 40 test trials. Within the test trials, each of the numbers appeared 10 times in a random order. The time between trials varied randomly from 1 to 3 s.

For Inspection Time, participants were exposed to a visual stimulus consisting of a pair of parallel vertical straight lines (connected with a horizontal bar at the top), one of which was markedly shorter than the other (Deary et al. 2004). The stimulus was displayed for varying durations, then immediately covered after offset by a visual pattern backward mask. The participants were asked to indicate the side (left or right) on which the longer line was present. The task does not involve the measurement of motor speed since there was no requirement for a fast response; the exposure time of the stimulus was varied and only the correctness of the response was used. The task consisted of 40 practice and 150 test trials with ten trials at each of 15 durations (6, 12, 19, 25, 31, 37, 44, 50, 62, 75, 87, 100, 125, 150 and 200 ms). The total number of correct responses was used as the measure of performance.

### General factor of information processing speed

In order to capture the variance shared by these five processing speed tests, *g*_speed_ was calculated using PCA. Only one component was indicated by scree slope analysis. Values of *g*_speed_ were calculated for each participant as a projection of the five processing speed tests’ loadings on the first unrotated principal component. In addition, residual, test-specific scores, namely the variance of each processing speed test not accounted for by *g*_speed_, were obtained from a confirmatory factor analysis of the five individual tests.

### Magnetic resonance imaging acquisition

All MRI data were acquired using a GE Signa Horizon HDxt 1.5 T clinical scanner (General Electric, Milwaukee, WI, USA) using a self-shielding gradient set with maximum gradient strength of 33 mT/m and an 8-channel phased-array head coil. The full details of the imaging protocol can be found in Wardlaw et al. (2011). Briefly, the DT-MRI examination consisted of 7 T_2_-weighted (*b* = 0 s mm^−2^) and sets of diffusion-weighted (*b* = 1000 s mm^−2^) single-shot spin-echo echo-planar (EP) volumes acquired with diffusion gradients applied in 64 non-collinear directions (Jones et al. 2002). Volumes were acquired in the axial plane, with a field-of-view of 256 × 256 mm, contiguous slice locations, and image matrix and slice thickness designed to give 2 mm isotropic voxels. The repetition and echo time for each EP volume were 16.5 s and 98 ms, respectively.

### Image analysis

All DT-MRI data were converted from DICOM (http://dicom.nema.org) to NIfTI-1 (http://nifti.nimh.nih.gov/nifti-1) format using the TractoR package for fibre tracking analysis (http://www.tractor-mri.org.uk). FSL tools (http://www.fmrib.ox.ac.uk/fsl) were then used to extract the brain, remove bulk motion and eddy current-induced distortions by registering all subsequent volumes to the first T_2_-weighted EP volume (Jenkinson and Smith 2001), estimate the water diffusion tensor and calculate parametric maps of FA from its eigenvalues using DTIFIT.

### Tract-based spatial statistics

Relationships between white matter FA, *g*_speed_, individual test scores and their residuals were investigated across the brain using TBSS (http://www.fmrib.ox.ac.uk/fsl). First, all participants’ FA volumes were linearly and non-linearly registered to a study-specific template. Second, a white matter skeleton was created from the mean of all registered FA volumes. This was achieved by searching for maximum FA values in directions perpendicular to the local tract direction in the mean FA volume. An FA threshold of 0.25 was applied to the skeleton to exclude predominantly non-white matter voxels. Third, for each subject’s FA volume, the maximum voxel perpendicular to the local skeleton direction was projected onto the skeleton. This resulted in one FA skeleton volume per subject corresponding to centres of white matter structures.

### Statistical analysis

Voxel-wise statistics were performed on the individual white matter skeletons using FSL’s ‘Randomise’ tool with 10,000 permutations; threshold-free cluster enhancement (TFCE) correction was applied to the resulting *p* value volumes to correct for multiple hypothesis testing. A corrected *p* value of less than 0.05 was considered statistically significant. All results are displayed as regions of significant voxels overlaid on the mean white matter skeleton, with the red/yellow colour scale given by thresholds set between 0.95 and 0.999 in FSLView.

A total of twenty-four linear regression models, of eight types, were fitted to the data to investigate relationships between FA in the white matter skeletons, childhood intelligence (age 11 IQ) and current measures of processing speed, controlling for age and sex. Equation (1) represents the first and simplest model

1 $${\text{FA}} = \beta_{0} + \beta_{1} \times {\text{age}} + \beta_{2} \times {\text{sex }} + \varepsilon,$$

where *β*_0_ is the intercept, *β*_1_ and *β*_2_ represent the coefficients of age and sex, respectively, and ε is the error term. Equations (2)–(8) describe the remaining model types, each of them predicting FA from age, sex and other variables of interest indexed by *β*_3_ and *β*_4_

2 $${\text{FA }} = \beta_{0} + \beta_{1} \times {\text{age}} + \beta_{2} \times {\text{sex}} + \beta_{3} \times {\text{age }}11{\text{ IQ}} + \varepsilon$$

3 $${\text{FA}} = \beta_{0} + \beta_{1} \times {\text{age}} + \beta_{2} \times {\text{sex}} + \beta_{3} \times g_{\text{speed}} + \varepsilon$$

4 $${\text{FA}} = \beta_{0} + \beta_{1} \times {\text{age}} + \beta_{2} \times {\text{sex }} + \beta_{3} \times {\text{individual test scores}} + \varepsilon$$

5 $${\text{FA}} = \beta_{0} + \beta_{1} \times {\text{age}} + \beta_{2} \times {\text{sex}} + \beta_{3} \times {\text{residual test scores}} + \varepsilon$$

6 $${\text{FA }} = \beta_{0} + \beta_{1} \times {\text{age}} + \beta_{2} \times {\text{sex}} + \beta_{3} \times g_{\text{speed}} + \beta_{4} \times {\text{age }}11{\text{ IQ}} + \varepsilon$$

7 $${\text{FA}} = \beta_{0} + \beta_{1} \times {\text{age}} + \beta_{2} \times {\text{sex}} + \beta_{3} \times {\text{individual test scores}} + \beta_{4} \times {\text{age }}11{\text{ IQ}} + \varepsilon$$

8 $${\text{FA}} = \beta_{0} + \beta_{1} \times {\text{age}} + \beta_{2} \times {\text{sex}} + \beta_{3} \times {\text{residual test scores}} + \beta_{4} \times {\text{age }}11{\text{ IQ}} + \varepsilon.$$

The individual processing speed tests (model types in Eqs. 4 and 7) were Digit-Symbol Substitution, Symbol Search, Simple Reaction Time, Four-Choice Reaction Time and Inspection Time described above. Since these individual processing speed tests were highly correlated, each was considered in a separate regression model. Similarly, residual test scores from each test (model types in Eqs. 5 and 8) were investigated separately. In all analyses, age in days at the time of scanning was used, and no model was tested that is not presented above.

## Results

Amongst the 730 participants who underwent MRI, 581 subjects (311 males) fulfilled the inclusion criteria of providing complete DT-MRI, childhood intelligence and processing speed data. The mean age at time of scanning was 72.7 ± 0.7 (range 71.0–74.2) years. Table 1 shows participant health characteristics for the whole sample, and male and female subgroups. In general, the male and female subgroups had fairly similar health characteristics, although males had significantly lower Mini-Mental State Examination scores, and significantly higher alcohol consumption, and self-reported incidence of diabetes and cardiovascular disease; conversely females had significantly higher incidence of arthritis and blood circulation problems. The majority of subjects had WMH loads rated on the two lowest Fazekas scores for both periventricular (67.5 %) and deep (78.8 %) white matter; females had a significantly higher Fazekas score in deep white matter compared with males.

**Table 1:** Participant health characteristics

	Total sample	Sex		*p* value
		Male	Female	
No. of subjects	581	311	270	
MMSE	28.85 (1.25)	28.73 (1.31)	28.98 (1.16)	>0.02
Education (years)	10.80 (1.14)	10.79 (1.17)	10.81 (1.10)	0.80
BMI (kg/m^2^)	27.88 (4.55)	28.03 (4.24)	27.72 (4.89)	0.41
Alcohol consumption (units)*	13.46 (14.19)	16.71 (15.44)	8.61 (10.36)	>0.001
Smoking status (%)				
Never	273 (47.0)	134 (23.1)	139 (23.9)	
Former	262 (45.1)	153 (26.3)	109 (18.8)	
Current	46 (7.9)	24 (4.1)	22 (3.8)	0.10
Hypertension (%)	288 (49.6)	162 (27.9)	126 (21.7)	0.19
Diabetes (%)	61 (10.5)	42 (7.2)	19 (3.3)	0.01
High cholesterol (%)	245 (42.2)	135 (23.2)	110 (18.9)	0.52
Cardiovascular disease (%)	161 (27.7)	105 (18.1)	56 (9.6)	>0.001
Arthritis (%)	273 (47.0)	125 (21.5)	148 (25.5)	>0.001
Blood circulation problems (%)	105 (18.1)	46 (7.9)	59 (10.2)	0.03
No. of subjects	575	308	267	
Fazekas score (0/1/2/3)				
Periventricular	17/371/150/37	9/199/84/16	8/172/66/21	0.58
Deep white matter	85/368/107/15	53/201/51/3	32/167/56/12	0.01

Measures given are mean (SD) for scalar data, and number of participants (percentage) for self-reported and WMH load dichotomous and ordinal data. WMH scores were generated from structural scans acquired as part of the LBC1936 imaging protocol by a trained neuroradiologist (Wardlaw et al. 2011). Differences between male and female subgroups were assessed using an independent samples *t* test for scalar data, and a χ^2^ test for dichotomous or ordinal data

*MMSE* Mini-Mental State Examination (maximum score 30), *BMI* Body mass index

* 446 (267 male) participants provide data on alcohol consumption

Mean and SD values for age 11 IQ, and the processing speed tests and their residuals are shown in Table 2. Males and females performed similarly on these tests, although females had significantly higher age 11 IQ and provided a significantly larger number of correctly decoded symbols in the Digit-Symbol Substitution test than males, while males produced a significantly greater number of correct responses in the Inspection Time task than females. As indicated in Table 3, Pearson’s product-moment correlations between the individual processing speed tests were moderate to large suggesting that a common factor could be extracted. As described above, PCA was then run on these data which produced a strong common factor, *g*_speed_, which explained 65 % of the total test variance; all cognitive tests had a large correlation with *g*_speed_, except Simple Reaction Time which had a moderate correlation.

**Table 2:** Participant mean (SD) values for age 11 IQ, and the information processing speed tests and their residuals

	Total sample	Sex		*p* value
		Male	Female	
No. of subjects	581	311	270	
Age 11 IQ	101.12 (15.21)	99.71 (16.43)	102.75 (13.51)	>0.02
Symbol Search	24.91 (6.14)	24.75 (6.28)	25.09 (5.97)	0.50
Digit-Symbol Substitution	57.04 (12.21)	55.01 (12.3)	59.38 (11.70)	>0.001
Simple Reaction Time (s)	0.27 (0.05)	0.27 (0.05)	0.27 (0.05)	0.53
Four-Choice Reaction Time (s)	0.64 (0.09)	0.64 (0.09)	0.64 (0.08)	0.96
Inspection Time	111.75 (11.15)	112.87 (11.39)	110.47 (10.74)	0.009
Residual SS	0.04 (3.67)	0.13 (3.71)	−0.06 (3.62)	0.53
Residual DS	0.16 (6.38)	−1.34 (5.97)	1.89 (6.41)	>0.001
Residual SRT (s)	6.88 × 10^−5^ (0.04)	−2.39 × 10^−3^ (0.04)	2.90 × 10^−3^ (0.05)	0.15
Residual CRT (s)	4.30 × 10^−4^ (0.05)	−2.89 × 10^−3^ (0.05)	4.26 × 10^−3^ (0.05)	0.11
Residual IT	0.11 (9.25)	1.56 (9.14)	−1.57 (9.10)	>0.001

‘Residual’ refers to variables that have been residualised for *g*_speed_

Differences between male and female subgroups were assessed using an independent samples *t* test

*SS* Symbol Search (units: number of rows inspected within the allocated time), *DS* Digit-Symbol Substitution (units: number of symbols decoded within the allocated time), *SRT* Simple Reaction Time (units: mean reaction time of correct responses in seconds), *CRT* Four-Choice Reaction Time (units: mean reaction time of correct responses in seconds), *IT* Inspection Time (units: number of correct responses)

**Table 3:** Pearson’s product-moment (*r*) correlations between the individual test scores and the general factor of information processing speed (*g*_speed_)

	SS	DS	CRT	SRT	IT
SS	–				
DS	0.6252	–			
CRT	−0.5011	−0.5565	–		
SRT	−0.256	−0.2489	0.4855	–	
IT	0.371	0.3976	−0.3842	−0.2641	–
*g*_speed_^*^	0.6843	0.8823	−0.5885	−0.3119	0.7742

*SS* Symbol Search (units: number of rows inspected within the allocated time), *DS* Digit-Symbol Substitution (units: number of symbols decoded within the allocated time), *SRT* Simple Reaction Time (units: mean reaction time of correct responses in seconds), *CRT* Four-Choice Reaction Time (units: mean reaction time of correct responses in seconds), *IT* Inspection Time (units: number of correct responses)

* These correlations are between each test and the component of which the test is a part. They thus give an indication of the loading of each test on the component

### Age and sex

**Fig. 1:**
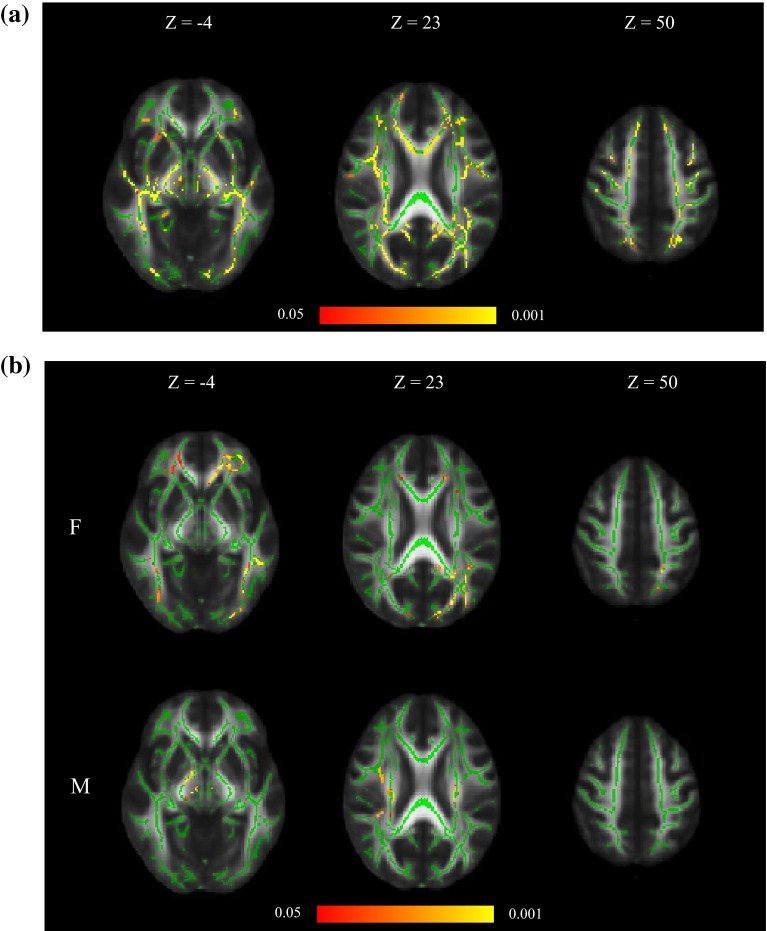
**a** Associations between white matter FA, age and sex (Eq. 1), **b** sex differences identified in the same model. Note that this pattern of significant voxels is similar to that observed for sex in all other models. In both figures, regions of significant voxels (*red*/*yellow*) are overlaid on the mean white matter skeleton (*green*). The effects were thresholded using TFCE at *p* < 0.05, with *yellow* indicating smaller *p* values

The first model (Eq. 1) investigated age and sex as the only predictors of white matter FA. Age, even in this narrow age sample, showed significant negative skeleton-wide associations with white matter FA (Fig. 1a). Sex effects were much less pronounced. Females had significantly higher FA than men, mainly in isolated parts of the genu and splenium of corpus callosum, whereas males had higher FA in small mid-brain segments of the superior corona radiata (Fig. 1b).

### Childhood intelligence

The second model (Eq. 2) investigated relationships between childhood intelligence and older age white matter FA. In this model with age and sex, childhood intelligence was not significantly correlated with white matter FA in any voxel.

### General factor of information processing speed

**Fig. 2:**
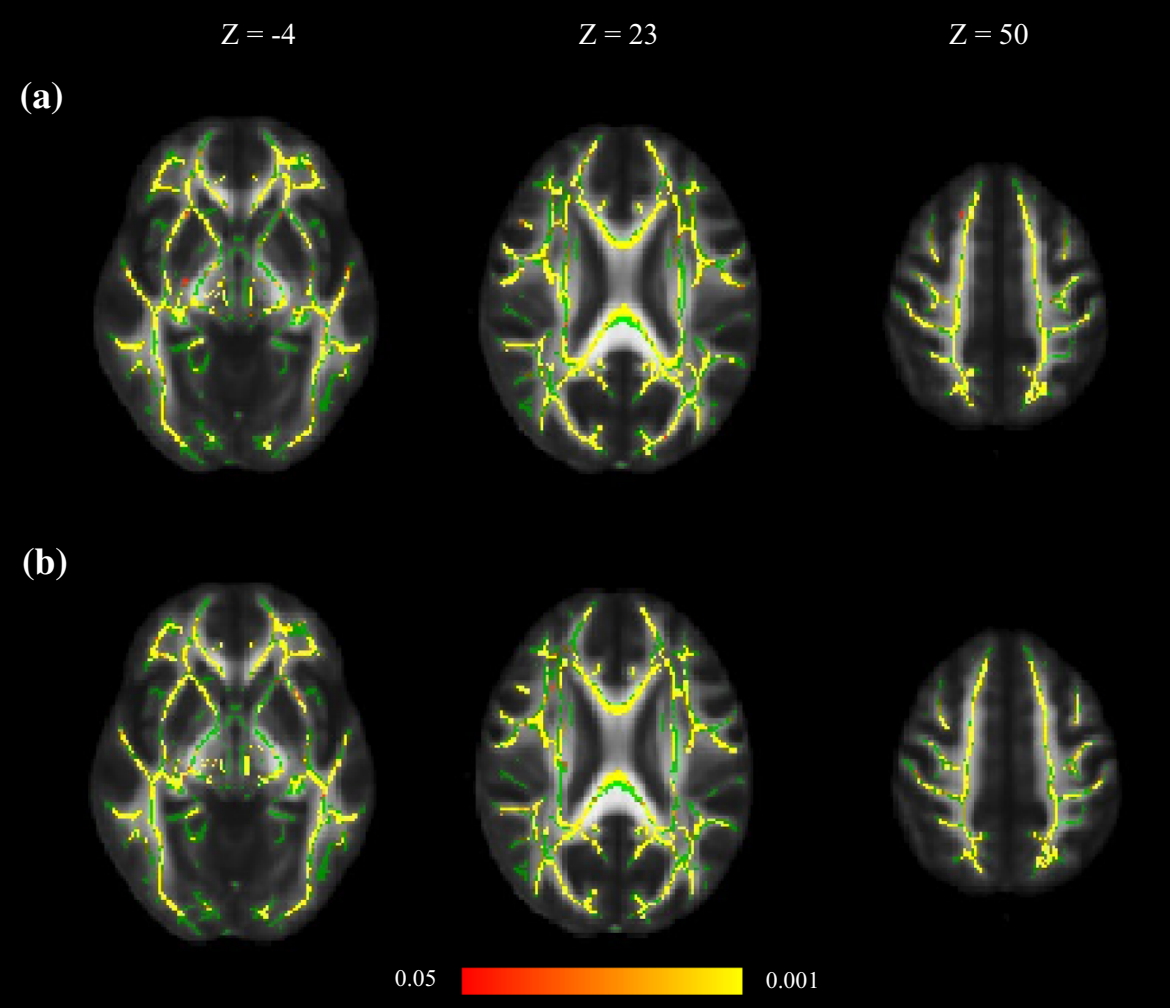
**a** Associations between white matter FA, *g*_speed_, age and sex (Eq. 3), **b** associations between white matter FA, *g*_speed_, childhood intelligence, age and sex (Eq. 6). Regions of significant voxels (*red*/*yellow*) are overlaid on the mean white matter skeleton (*green*). The effects were thresholded using TFCE at *p* < 0.05, with *yellow* indicating smaller *p* values

Results from the third model (Eq. 3) showed that the general information processing speed factor, *g*_speed_, had significant positive skeleton-wide associations with white matter FA after controlling for age and sex (Fig. 2 upper panels). This association remained (and was not significantly weakened) when childhood intelligence was added as a further covariate (Eq. 6; Fig. 2 lower panels). With *g*_speed_ included in the model, age was no longer significantly associated with white matter FA; the association with sex remained.

### Individual tests of information processing speed

**Fig. 3:**
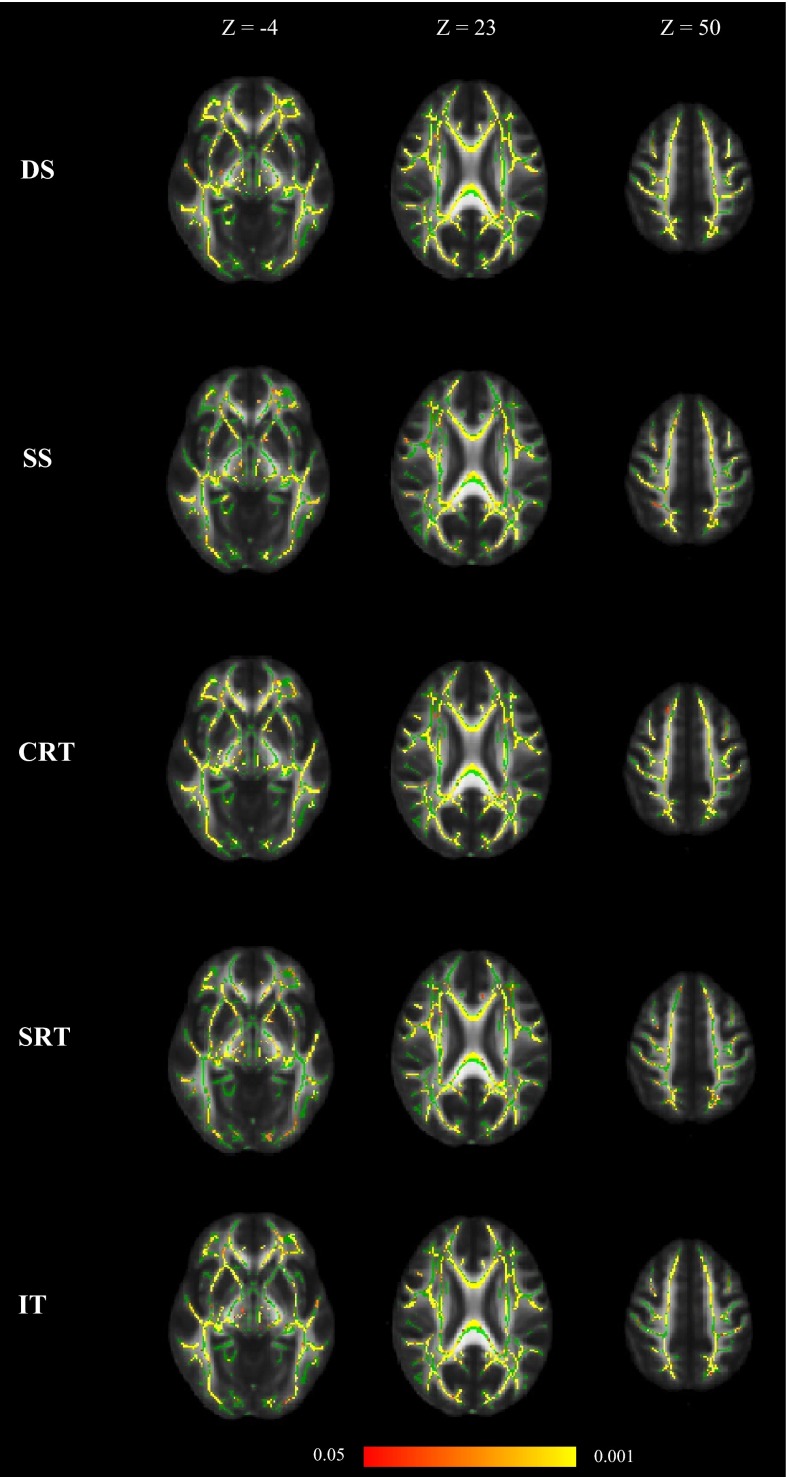
Associations between white matter FA, individual information processing speed tests, age and sex (Eq. 4); *DS* Digit-Symbol Substitution, *SS* Symbol Search, *CRT* Four-Choice Reaction Time, *SRT* Simple Reaction Time and *IT* Inspection Time. Regions of significant voxels (*red*/*yellow*) are overlaid on the mean white matter skeleton (*green*). The effects were thresholded using TFCE at *p* < 0.05, with *yellow* indicating smaller *p* values

All five individual processing speed tests were significantly associated with white matter FA across the white matter skeleton after controlling for age and sex (Eq. 4; Fig. 3). Higher scores on Digit-Symbol Substitution, Symbol Search and Inspection Time were positively correlated with white matter FA, whereas faster (lower) Simple and Four-Choice Reaction Time were negatively correlated. The directions of association were therefore all consistent with higher FA being associated with faster processing speed. Age was also significantly negatively associated with white matter FA across the white matter skeleton in all models, apart from with Four-Choice Reaction Time, where the age effect was attenuated to non-significance. Sex differences were evident in all models apart from those including Simple and Four-Choice Reaction Time, where sex did not significantly contribute to explaining the variance in FA. When childhood intelligence was added as a covariate (Eq. 7), it did not significantly contribute to explaining the variance in FA in any of the models, but did slightly decrease the significance with which Symbol Search and Inspection Time related to FA. Age remained a significant predictor of white matter FA at age 73 for all five processing speed tests.

### Residual analysis of individual cognitive tests contributions

**Fig. 4:**
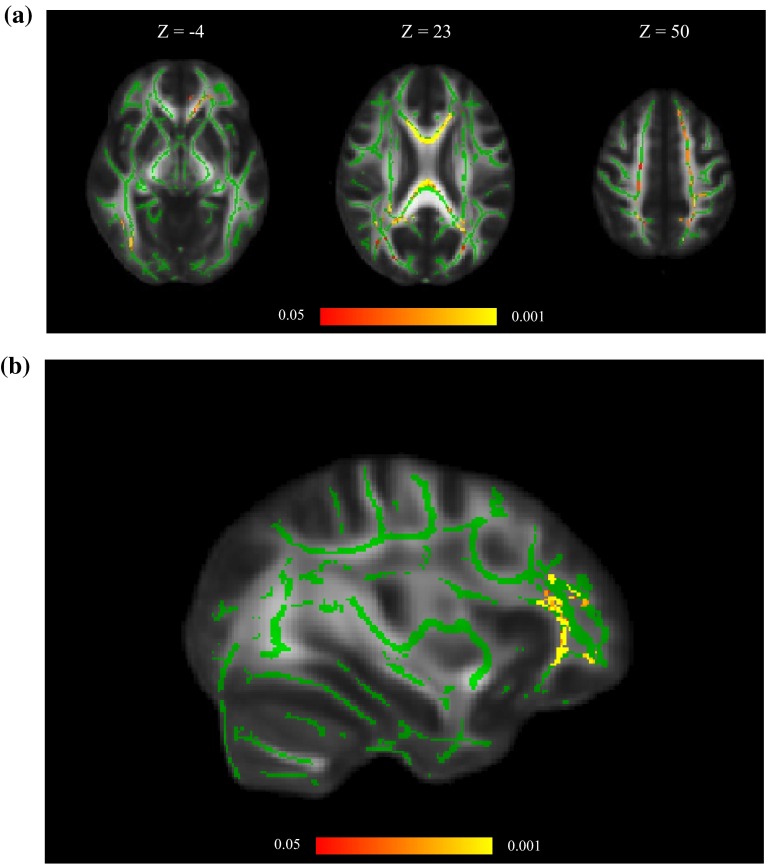
**a** Associations between white matter FA, residual Four-Choice Reaction Time, age and sex (Eq. 5), **b** associations between white matter FA, residual Digit-Symbol Substitution, age and sex (Eq. 5). Regions of significant voxels (*red*/*yellow*) are overlaid on the mean white matter skeleton (*green*). The effects were thresholded using TFCE at *p* < 0.05, with *yellow* indicating smaller *p* values

**Fig. 5:**
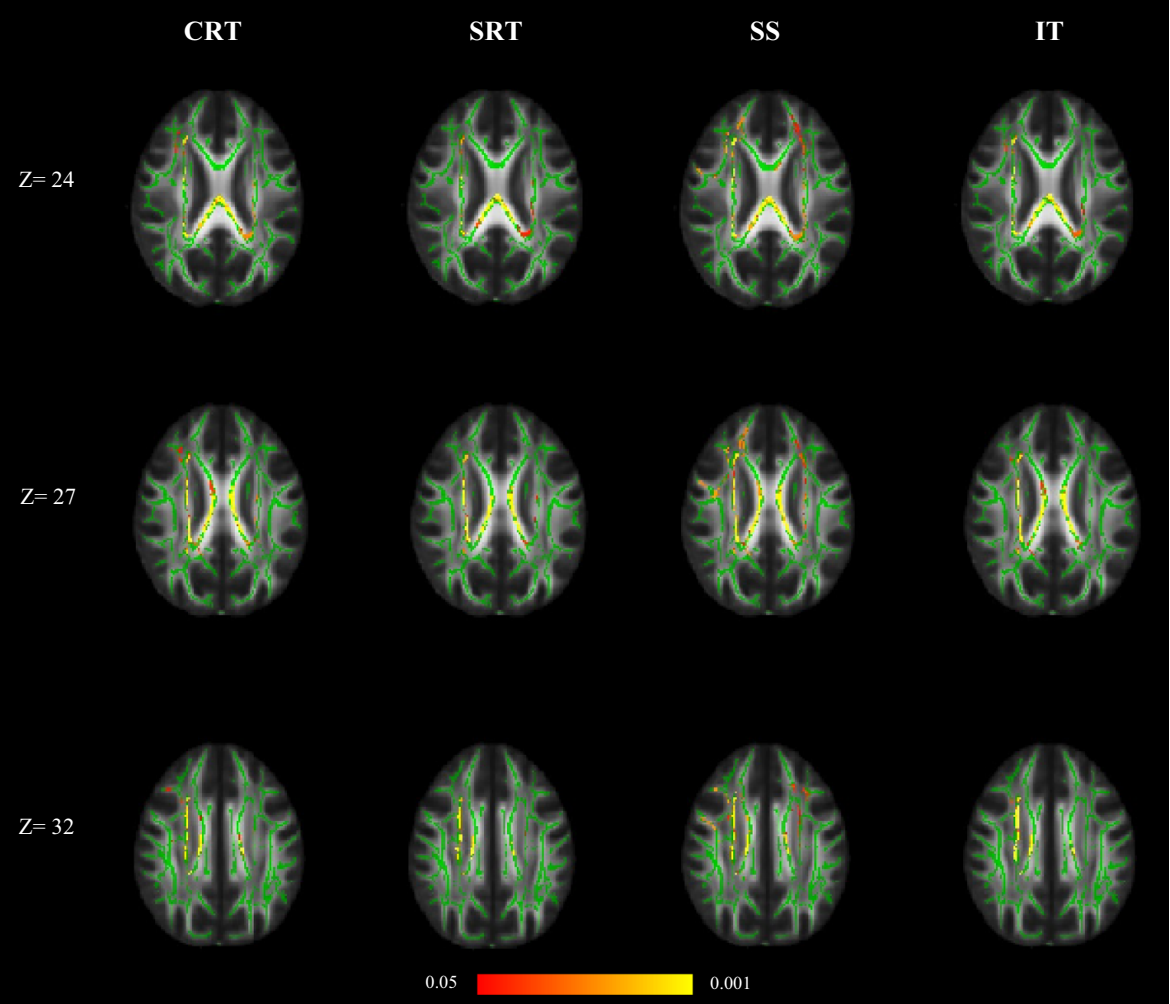
Associations between white matter FA, childhood intelligence, age, sex and residual (*left* to *right*) Four-Choice Reaction Time (CRT), Simple Reaction Time (SRT), Symbol Search (SS) and Inspection Time (IT) (Eq. 8). Regions of significant voxels (*red*/*yellow*) are overlaid on the mean white matter skeleton (*green*). The effects were thresholded using TFCE at *p* < 0.05, with *yellow* indicating smaller *p* values

Finally, we analysed the residuals of each processing speed test after removing the shared variance provided by *g*_speed_ and additionally controlling for age and sex (Eq. 5). This model asks whether the specific cognitive capabilities associated with each processing speed task, stripped of that task’s association with *g*_speed_, is associated with FA. Figure 4a shows that residual Four-Choice Reaction Time was significantly negatively associated with FA predominantly in the genu of corpus callosum. Residual Digit-Symbol Substitution was significantly positively associated with FA more generally in frontal white matter (Fig. 4b). Residual Symbol Search, Simple Reaction Time and Inspection Time did not have significant associations with FA; that is, their associations with FA were accounted for by the variance these tests shared with other processing speed tests. When childhood intelligence was added as a covariate (Eq. 8), it had a significant positive association with FA principally in the splenium and posterior body of corpus callosum in all residual models apart from Digit-Symbol Substitution where there was no association (Fig. 5). Age remained a significant predictor of white matter FA in all models.

## Discussion

The present study used voxel-based methods to extend previous analyses that have employed quantitative tractography to investigate links between cognitive ability and white matter structure in the LBC1936 (Penke et al. 2010, 2012). The results showed that, in older age, there were skeleton-wide associations between white matter FA and measures of processing speed which supports the hypothesis that the latter is not localised to specific tracts (Haász et al. 2013; Penke et al. 2010, 2012). This was the case regardless of whether individual tests or *g*_speed_ was used to index processing speed. There were, however, some specific areas of the brain that were associated with variance in some tasks that were not part of general processing speed. Finally, intelligence assessed at age 11 years was not associated with white matter FA in most models suggesting that older age associations between white matter structure and processing speed are generally not accounted for by cognitive ability measured in youth. Our findings highlight the crucial role white matter plays in providing a neuroanatomical structure for individual differences in the information transfer underpinning fundamental aspects of human intelligence. They also provide support for viewing the brain as a highly connected distributed network with the slower processing speed found in older age being part of a process of cortico-cortical disconnection (Bennett and Madden 2014; Felleman and Van Essen 1991; Sporns 2013).

Beyond *g*_speed_, we investigated whether the individual processing speed tests had specific associations with white matter FA. This was achieved by removing the shared variance with all the other tests from each individual test by residualising for *g*_speed_. The residuals of Symbol Search, Simple Reaction Time and Inspection Time were no longer associated with white matter FA, indicating that variance specific to these tests was not related to white matter structure beyond their shared variance with *g*_speed_. However, residual Four-Choice Reaction Time was significantly associated with FA in the genu of corpus callosum, and residual Digit-Symbol Substitution was associated with FA in frontal white matter. This may be due to these tasks’ overlap with cognitive abilities other than information processing speed. For example, there is evidence that the Digit-Symbol Substitution task also involves use of memory which is associated with frontal fibres (Charlton et al. 2010; Piccinin and Rabbitt 1999). Similarly, faster responses on Four-Choice Reaction Time require inter-hemispheric coordination and selection of manual responses, which is associated with the corpus callosum (Doron and Gazzaniga 2008).

Childhood intelligence was not significantly associated with older age white matter FA in the model with age and sex as the only covariates. Similarly, there were no significant associations between childhood intelligence and white matter FA when *g*_speed_, or any of its individual processing speed tests, were also included in the model. However, when the same analysis was repeated with residual processing speed scores, childhood intelligence became significantly associated with white matter FA in the splenium and posterior body of corpus callosum for all residual scores except Digit-Symbol Substitution. These data therefore suggest that although early life intelligence does not strongly predict later life global white matter FA, it does predict the specific relationship between splenium FA and better scores on the *g*_speed_-independent components of these individual cognitive tests. A possible explanation for this finding is that the residual test scores reflect other orthogonal cognitive domains to processing speed, such as memory, which are known to relate to measures of childhood cognitive ability (Deary 2014). These results should be considered in the context of other analyses performed in the LBC1936, for example regarding WMH burden and brain volume, which show that early life cognitive function has a significant effect on white matter structure in older age. Specifically, Valdés Hernández et al. (2013) found in approximately 600 LBC1936 participants that childhood intelligence was negatively correlated with WMH volume (*r* = −0.08) suggesting that lower cognitive ability in youth leads to increasing white matter damage with age, while Royle et al. (2013) reported that childhood intelligence was a predictor of estimated prior (*r* = 0.12) and measured current brain volume (*r* = 0.11) in older age.^1^ However, Penke et al. (2012) found that white matter FA measured in 12 major tracts using quantitative tractography was more associated with intelligence in later rather than early life, as reported here. It is therefore possible that DT-MRI biomarkers, even measured skeleton-wide in voxel-based analyses, reflect the effects of cognitive ageing more than they reflect lifelong-stable differences in intelligence. Clearly, the availability of measures of childhood cognitive ability is an uncommon feature of the current study which makes generalising these findings more difficult; it would be tempting to conclude that early life cognition need not be a key feature of DT-MRI studies of normal ageing. Further work, in both cross-sectional and longitudinal analyses, in cohorts with such early life cognitive data, for example the Aberdeen Birth Cohorts (http://www.abdn.ac.uk/birth-cohorts), is required to clarify this finding.

Our results showed some subtle sex differences: females had higher white matter FA in isolated parts of the genu and splenium of corpus callosum, whereas males had higher white matter FA in small mid-brain segments of the superior corona radiata. This may reflect differential brain ageing according to sex, although males and females had fairly similar healthy characteristics in this cohort. These sex differences in white matter structure will require replication in other large, later life cohorts.

This study has several strengths compared to previous studies of information processing speed and white matter structure in older age. The LBC1936 is a large cohort with a narrow age range and generally healthy participants. Subjects took five processing speed tasks drawn from different traditions, namely psychometric, experimental psychological and psychophysical tests. Together, they created a reliable measure of *g*_speed_. Furthermore, the availability of childhood intelligence data from age 11 allows prior cognitive ability to be controlled for and thus permits changes that are specifically due to ageing to be identified more efficiently. This is an important issue since we have shown previously that all of the processing speed tests are strongly associated with general cognitive ability from youth, about 60 years earlier (Deary et al. 2010). Analysis of the DT-MRI data using TBSS allows relations between processing speed and white matter structure to be investigated voxel-by-voxel across the brain, rather than in a few major tracts, in an automated and user-independent fashion. However, this method only allows comparison of individual differences in white matter FA across those areas which show good spatial concordance with the white matter skeleton. Thus, local gyral white matter and other areas, which show a high degree of morphological variability, were not included in these analyses.

The narrow age range of this sample can, conversely, also be viewed as a limitation because our findings cannot be generalised to younger populations and are only representative of older subjects. Similarly, because the LBC1936 subjects are generally healthy, our finding cannot be directly applied to ageing populations with decreased processing speed due to neurodegenerative diseases, such as dementia. In order to keep the number of models manageable, we did not investigate associations between processing speed test scores and other DT-MRI biomarkers, such as mean, radial and axial diffusivity; these variables may be more prone to partial volume CSF-contamination errors than FA (Metzler-Baddeley et al. 2012). Finally, we did not take into account WMH; how these features might affect the results reported here is currently being investigated in the subgroup of LBC1936 participants who have significant lesion burden.

In conclusion, we found using voxel-based analyses that *g*_speed_, as well as five varied tests of information processing speed, had significant skeleton-wide associations with white matter FA. These results indicate that quicker and more efficient information processing requires global connectivity in older age. Conversely, they can also be interpreted to imply that there is an age-related global reduction in connectivity that affects all networks, including those associated with the administered processing speed tests. Childhood intelligence was not associated with white matter FA, except for several residual (*g*_speed_-independent) test score models, suggesting that associations between white matter structure and information processing speed measured in older age are not generally accounted for by cognitive ability measured in youth.

## Footnotes

1 Although not involving brain white matter structure, another LBC1936 analysis also emphasised the importance of having cognitive test data from early life. Specifically, Karama et al. (2014) found that widespread cross-sectional associations at age 73 years between brain cortical thickness and cognitive ability were wholly confounded by cognitive ability tested at age 11.
